# Variable pollen viability and effects of pollen load size on components of seed set in cultivars and feral populations of oilseed rape

**DOI:** 10.1371/journal.pone.0204407

**Published:** 2018-09-20

**Authors:** Åsa Lankinen, Sandra A. M. Lindström, Tina D’Hertefeldt

**Affiliations:** 1 Plant Protection Biology, Swedish University of Agricultural Sciences, Alnarp, Sweden; 2 Biodiversity, Department of Biology, Lund University, Lund, Sweden; 3 Department of Ecology, Swedish University of Agricultural Sciences, Uppsala, Sweden; 4 Swedish Rural Economy and Agricultural Society in Scania, Kristianstad, Sweden; Duzce Universitesi, TURKEY

## Abstract

Pollination success is important for crop yield, but may be cultivar dependent. Less is known about which floral traits influence pollination success. Floral traits, e.g. traits related to attraction and reward, can also contribute to gene flow via pollen, the latter being of particular importance in oilseed rape (*Brassica napus*) where gene flow occurs between plants of crop, volunteer and feral origin as well as related taxa. We investigated the relationship between pollen load size and seed set in winter oilseed rape. We compared variability in pollen-viability traits, flower production (flowers from the main raceme times number of branches) and seed number and weight per siliqua among cultivars and feral populations (growing outside of agricultural fields) under controlled conditions. Both seed number and weight were saturated at relatively low pollen loads in the tested cultivar. Pollen viability and estimated flower production differed among cultivars, indicating that these traits could contribute to yield variability. Seed weight per siliqua, but not pollen traits or flower production, was lower in ferals compared to cultivars. Thus, while the probability of establishment may be reduced in ferals (due to lower seed weight per siliqua) this will not necessarily impact their contribution to gene flow via pollen. In oilseed rape a relatively low pollen load may be sufficient for full seed set in some cultivars, suggesting less dependence on insect pollination for high yield than generally expected. Our results also showed that previously less investigated floral traits, such as pollen viability, pollen tube growth rate and flower number, can differ between cultivars. Studies of these traits may provide targets for increasing crop yield and provide general knowledge about gene flow between cultivated, feral and related wild populations.

## Introduction

Ecological intensification is proposed as a means to ameliorate agricultural production through utilization of ecosystem services such as insect pollination [1]. Insect pollination is vital for high yields of many food crops [2]. However, in many crops detailed knowledge of pollination biology is scarce [2]. For example, studies on winter oilseed rape (*Brassica napus* L.) have shown highly inconsistent effects of insect pollination on yield [3]. Dissimilar effects among cultivars regarding benefits of insect pollination on yield were recently shown [4,5], but little is known about how plant traits of cultivars contribute to differences in pollination success.

Improved knowledge on plant traits influencing pollination success could be essential for future plant breeding and improved crop management [6]. Variation among insect-pollinated cultivars of various crops have been evaluated with respect to differences in self-incompatibility [7], nectar secretion [8], corolla tube length [9], size and number of flowers [8], and time of flowering [10]. However, few studies have explored differences among modern cultivars of a crop in relation to particular floral traits that may be of importance for efficiency of pollen transfer or influence pollination success, i.e. lead to high seed set (but see e.g., [11]).

It is well known that floral traits, such as flower size, shape, colour, scents and nectar content can strongly influence pollinator visitation rates, number of pollen grains transferred between flowers, and seed production [12]. However, not only floral traits that attract pollinators and give floral rewards are important for seed yield. Pollen germination rate on the stigmatic surface and pollen tube growth rate in the style can be major determinants of siring success [13]. Pistil traits (e.g. large stigmatic surface and long style) can enhance competition among pollen growing in the style, which in turn can increase quantity or quality of seeds. The relationship between pollen load size and seed quantity and quality can be described by “filling”, “sorting” and “surplus” stages, where number of seeds is assumed to increase in the filling stage while seed quality (i.e. fitness-related seed traits) rather is assumed to increase in the sorting stage because of pistil selection of high quality pollen during pollen competition [14]. Large pollen loads can lead to stigma clogging and lowered seed set [15]. Thus, it is important to determine the relationship between pollen load size and components of seed yield in order to fully understand the significance of pollination success for seed yield.

The availability of multiple cultivars provides plant material to test for variability in pollen viability traits, flower production and nectar volume in order to determine the underlying mechanisms of effects of insect pollination on yield, and possible causes to differences among cultivars. Moreover, including feral plants from various locations in such comparisons provide an opportunity to learn about future containment of gene flow via pollen in *B*. *napus* [16]. Gene flow in *B*. *napus* occurs between crop, wild and feral relatives [17]. Crop seeds that are dispersed to seminatural habitats can function as a source for genetic recombination, gene stacking and genotype evolution, while long-term seed dormancy allows for build-up of a volunteer seedbank [18–20]. As a result, *B*. *napus* volunteers occur with stacked traits and certified seed lots with traces of other cultivars [21–23]. While mass-flowering crops have been bred for optimal seed production and may benefit from synchronous flowering to attract pollinators, feral plants have to endure more resource-poor conditions as well as a lack of an advantage of mass display. Studies of gene flow from oilseed rape to its wild relative *Brassica rapa* have shown that the number of introgressed generations and environmental conditions may either have no effect on hybrid fitness, or increase hybrid fitness [19,24].

In this study, our aim was to gain knowledge of potentially significant factors for pollination success and gene flow in winter oilseed rape cultivars and ferals. Oilseed rape cultivars are bred in two different ways. The ‘open pollinated’ types are bred by traditional pedigree techniques. Since 1990, ‘hybrid’ types are bred by crossing two open pollinated cultivars. This technique utilizes a heterosis effect in the seeds, which could lead to more vigorous plants with better stress tolerance. Documented differences among oilseed rape cultivars include for example yield and days to maturity [25]. First, we evaluated the relationship between pollen load size and number of seeds and seed weight per siliqua (i.e. assessments of seed quantity and quality) using controlled hand-pollinations in one cultivar (Compass). We hypothesized that the relationship between pollen load size and seed production is described by non-linear functions with diminishing returns, where seed quantity should reach a saturated value faster than seed quality, provided that higher levels of pollen competition increase seed quality [14]. Secondly, we measured variability in pollen-viability traits and in nectar, flower and seed production in seven cultivars and in five feral populations all grown in a uniform environment (common garden or greenhouse). Both open pollinated and hybrid cultivars were included because recent studies suggest that open pollinated cultivars increase in yield in the presence of pollinators, while hybrids do not [4,5]. We hypothesized differences among cultivars. We also hypothesized lowered fitness in ferals compared to in cultivars. Impaired pollen viability, nectar and flower production in ferals could directly reduce gene flow from ferals to cultivars, while low seed set and low seed weight could rather influence the probability of feral establishment [26].

## Materials and methods

### Species description

Oilseed rape flowers (*Brassica napus*) are protogynous and consist of one stigma, four nectaries and six stamens (four longer that dehisce outwards, and two shorter that dehisce inwards) [27]. Flowering begins at the main stem, on the lowest bud. A flower is open and receptive to pollen for three days [27]. Pollen grains are sticky, heavy, and large (25 μm), and are lumped together with pollen kit, counteracting spread by wind [28]. Pollen longevity is at least 3 days and reported to be up to 15 days [29]. Flowers develop into siliqua, containing up to 17–32 seeds [30].

Oilseed rape is self-compatible to 50% or more (outcrossing range 12–55%, [31]), and cross-pollination occurs via wind, insects or contact between plants [32]. The efficiency of wind pollination is low and unspecific compared to insects [32]. The oilseed rape flower is visited by a variety of pollinating insects; honey bees, bumble bees, other bees, hover flies, marsch flies and other flies [5,10].

Seeds of *B*. *napus* are primarily non-dormant but adverse conditions such as darkness and low temperature can induce secondary dormancy [21]. The inducible dormancy in combination with seed loss at harvest due to silique shattering enable seed bank formation and subsequent occurrence of volunteer and feral plants [18,21]. Spilled oilseed rape seeds readily germinate in natural and semi-natural habitats, forming feral populations [33]. In a long-term study in agricultural areas in four European countries, feral populations were found in all areas [19].

### Collection and cultivation of plant material

In early spring (April) 2011, winter oilseed rape seedlings (*n* = 14–16 per cultivar) were collected from agricultural fields in southwest Scania, Sweden (S1 Table). We selected seven cultivars. Three were bred with open pollination methods; Galileo (Lantmännen SW Seed AB, Svalöv, Sweden), Vision (Lantmännen SW Seed AB, Svalöv, Sweden) and Catalina (Monsanto, Creve Coeur, USA). Four were bred with hybrid breeding methods; Visby (Norddeutsche Pflanzenzucht Hans-Georg Lembke KG, Hohenlieth, Germany), Excalibur (Monsanto, Creve Coeur, USA), Abakus (Norddeutsche Pflanzenzucht Hans-Georg Lembke KG, Hohenlieth, Germany) and Compass (Deutsche Saatveredelung AG, Issum, Germany). All hybrids had restored male fertility. We selected the open pollinated cultivars Galileo and Catalina, and the hybrids Excalibur and Compass because they were evaluated in relation to benefits of insect pollination in previous studies [4,5]. The additional cultivars were added from available cultivars grown in the area.

In addition to the oilseed rape cultivars, seedlings from five feral oilseed rape populations (*n* = 4–9 per population) were collected from field margins of arable land (S1 Table).

Cultivated and feral plants were planted in five liter pots with potting compost mixed with slow-release Osmocote fertilizer and placed in a common garden at the Department of Biology, Lund University from April until the end of the experiment. The majority of plants survived this procedure (< five plants died). We investigated differences in height and developmental stage between cultivated and feral plants at the time of potting. The developmental stage was quantified according to Leuchovius and Sturesson [34].

The plants were protected by a coarse net to avoid damage by birds but allowing for pollination visits. During the summer, we measured pollen viability and pollen tube growth rate, and components of flower- and seed production in cultivars and feral plants (S1 Table).

A subset of plants from the cultivars was placed in an insect-free greenhouse, allowing us to conduct crosses in the absence of pollinators and assess nectar production (secretion volume). The pots were widely spaced in the greenhouse to create good light conditions for side branch formation. In cultivars, we also evaluated environmental effects on pollen traits, plant size, components of flower-, and seed production, as these traits were measured in both common garden and greenhouse (S1 Table, S1 Appendix).

### Hand-pollination experiment

To determine the relationship between pollen load size and seed production per siliqua, we conducted controlled hand-pollination on emasculated flowers of the greenhouse-grown cultivar Compass (CH) (S1 Table). We chose this hybrid because it was part of a previous study showing that hybrids did not benefit from increased insect pollination [5]. We here developed the technique of hand-pollination with a controlled amount of pollen with cv. Compass as a pilot cultivar to investigate the amount of pollen grains needed for full seed set. Flowers selected on side branches were emasculated at flower opening in order to avoid self-pollination. On each of five plant individuals we made eight crosses that differed in pollen load size, using 25, 50, 100, 200, 300, 400, 500 or > 500 pollen grains. Crosses were performed by mixing pollen from all anthers from two flowers, obtained from two pollen-donating plants, on a microscope slide. To determine pollen load size, pollen was counted under a dissecting microscope, except for the > 500 pollen load treatment where as much pollen as possible was added in several layers. Pollen was transferred to the stigma directly from the slide by gently dipping the stigma in the pollen. For pollen loads containing up to 100 pollen grains, we judge that our estimates were as accurate as ± 5 pollen grains, while for larger pollen loads it rather reached ± 25 pollen grains.

Seed quantity and quality following hand-pollination were measured as the number of seeds per siliqua (= quantity) and seed weight per siliqua (= quality). We compared seed production in hand-pollinated flowers with that of control flowers from both greenhouse-grown (autonomously selfed) and common garden-grown (open-pollinated) plants of the cultivar Compass.

### Measurements of pollen traits

As an indication of pollen viability and pollen competitive ability in cultivars and feral populations, we assessed pollen germination rate and pollen tube growth rate *in vitro* by germinating pollen in Hoekstra medium [35], with 16% sucrose. Bosac *et al*., [36] suggested that 17% sucrose was optimal for pollen germination of oilseed rape. Because they used a different germination medium, we tested a range of sucrose concentrations (between 15 and 25%) and found optimal germination at 16%. Pollen grains from two flowers per plant were sprinkled onto a drop of the medium on a microscopic slide. The two flowers were of different age. The microscopic slides were placed in a dark chamber with a temperature of ca 20–21°C for 3 h. Pollen germination was terminated by adding 100% glycerol. Pollen germination rate was determined under a light microscope as the percentage of germinated pollen grains from ca 100 pollen grains in a randomly chosen area. Pollen tube growth rate was estimated by measuring the length of ten pollen tubes per sample. In order to minimize environmental influence [37], all pollen germinations were conducted at approximately the same time every day over a two-week period.

We germinated pollen from 7–9 plants per cultivar (*n* = 7)/feral population (*n* = 5) (or less when fewer feral plants were available, see S1 Table) and determined pollen germination and pollen tube growth rate in each sample. Pollen from a maximum of 20 plants was germinated in the same batch, containing both cultivated and feral plants grown in the common garden as well as cultivated plants grown in the greenhouse (see S1 Appendix). We also confirmed repeatability of pollen trait measurements among individual plants (suggesting a genetic influence on this trait) as well as an influence of environmental factors by repeated measured of some individuals in different temperatures and environments (see S1 Appendix, S2 Table). Pollen from most plants was germinated once (or occasionally twice). For comparisons among cultivars and feral populations we used the average when several pollen germinations per plant were performed. In an additional set of pollen germinations we confirmed that age of pollen (newly open flowers, ca one day old vs. older flowers with all anthers open) had no influence on pollen traits (S1 Appendix).

### Measurements of nectar production

Nectar production (amount) was measured on all plants grown in the pollinator-free greenhouse (*n* = 50, only cultivars) (S1 Table). We collected nectar using micro-capillary tubes (1μl) (cf. [38]) on three flowers per plant. Nectar volume per sample was determined by estimating the proportional length of the tube filled with nectar.

### Measurements of components of flower and seed production

We selected flower production and seed production per siliqua as sporophytic fitness traits, as these traits are important components of total seed production. We estimated flower production by multiplying number of flowers on the main raceme with number of branches, hereafter denoted “estimated flower production”. Seed production per siliqua was assessed as i) length of siliqua, ii) number of seeds and iii) seed weight per siliqua, using the average of ten healthy looking silique per plant individual, chosen from the whole plant. It should be noted that these estimates only represent components of total flower and seed production. Estimated flower production has previously been used in wild study systems as an indication of plant size [39], which can be an important trait for pollinator preference. We chose to evaluate seed traits per siliqua as this component of seed production is more closely related to pollen limitation and pollen viability. We estimated three seed traits to be able to investigate potential trade-offs between number of seeds and seed weight. After peak flowering, plants in the common garden were brought into the greenhouse, allowing seeds to ripen in a controlled environment.

### Statistics

We fitted data from our controlled hand-pollination experiment to likely non-linear functions (inverse, S-shaped and quadratic), because we expected the relationship between pollen load size and seed production to be described by a non-linear function [14]. We fitted separate curves for i) number of seeds per siliqua and ii) seed weight per siliqua.

We performed two types of analyses to test whether pollen traits and sporophytic fitness traits differed i) among cultivars (denoted analysis 1) and ii) between cultivars and feral populations (denoted analysis 2). We used various analyses of variance (mainly nested ANOVA and mixed-model ANOVA) in SPSS [40]. Non-significant interactions (*P* > 0.20) were excluded, and the model was re-run. Significant factors were examined by using Tukey HSD post-hoc test. In models investigating differences among the seven cultivars (analysis 1), we included the factors breeding method (open pollinated or hybrid, fixed factor) and cultivar (fixed factor, as we were interested in assessing differences among these specific cultivars). Cultivar was nested under breeding method, i.e. cultivars were compared within each of the two subgroups of open-pollinated vs. hybrid cultivars. For tests of both the seven cultivars and the five feral populations (analysis 2) we included category of plant (cultivated or feral) as a fixed factor and cultivar/feral population as a random factor (as in this case we were mainly interested in the general differences among any cultivars/populations). Cultivar/feral population were nested under category of plant. For the models with pollen tube growth rate as dependent variable, we included pollen germination rate as a continuous covariate.

Because all three seed traits per siliqua (siliqua length, seeds per siliqua and seed weight per siliqua) were correlated (*r* = 0.557–0.589, *n* = 98), we chose to use only seed weight per siliqua in all analyses with one exception. We selected this trait as we assumed it to be the best predictor of seed yield. One exception concerned the analysis on environmental effects on seed production, where the number of seeds per siliqua was included, as we were interested in potential effects on both seed quantity and seed quality in this case (see S1 Appendix). For nested ANOVA:s involving analysis 1 and 2 on fitness traits (estimated flower production and seed weight per siliqua) we pooled data from both common garden and greenhouse to ensure a balanced design, as there was missing data (resulting in a low number of replicates) for some cultivars in the common garden. These traits did not differ between environments (see S1 Appendix).

Pollen germination rate was rescaled as an arcsine transformed proportion to fulfil assumptions of homogeneity of variance and normality. All other variables were normally distributed. Type III Sum of Squares were used in all ANOVAs.

## Results

### Relationship between pollen load size and seed production per siliqua

**Fig 1:**
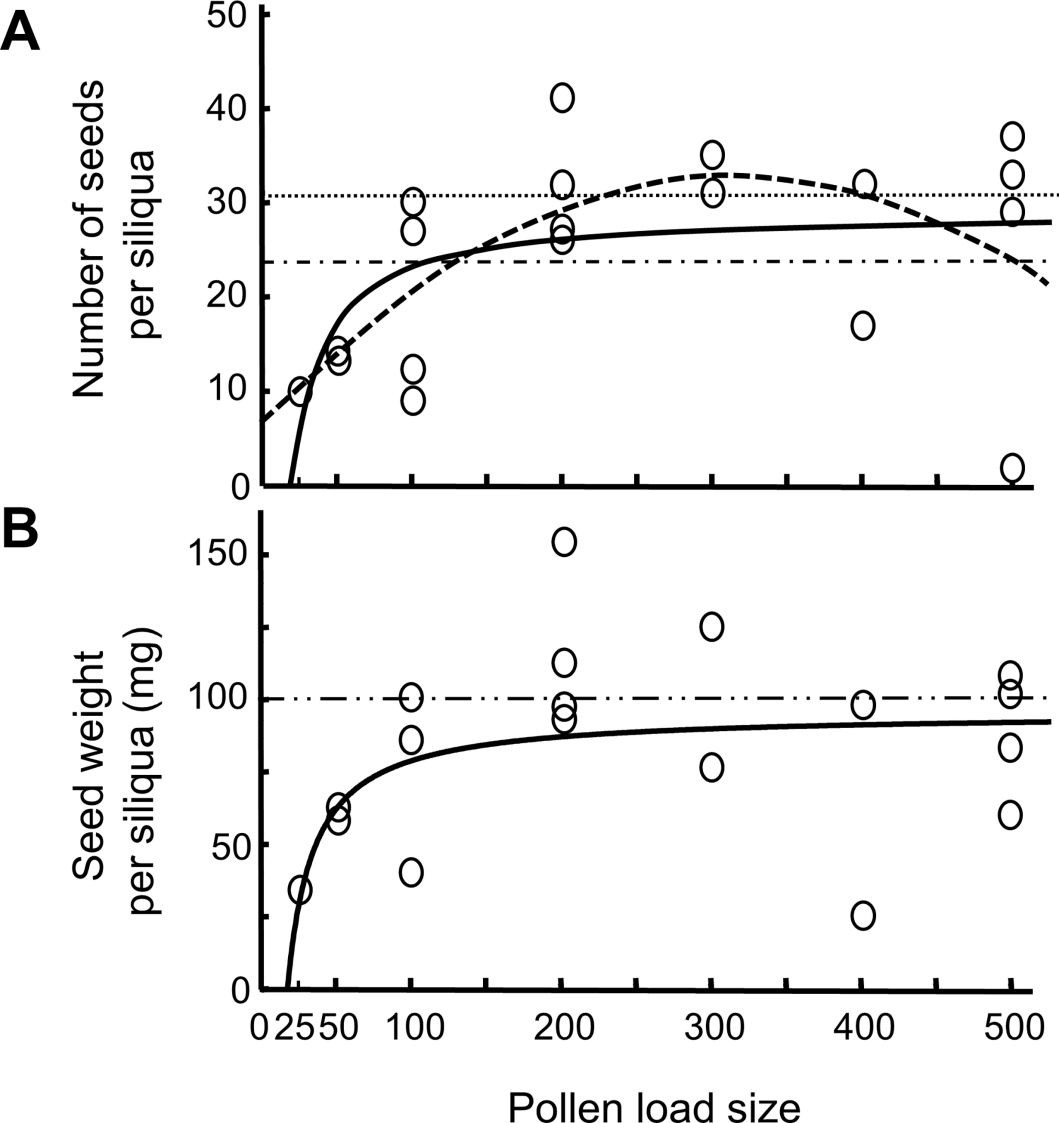
Relationship between pollen load size and seed traits in winter oilseed rape cultivar Compass (CH). **(A)** Number of seeds per siliqua and **(B)** seed weight per siliqua following hand-pollination with pollen load sizes of 25, 50, 100, 200, 300, 400 and 500 pollen grains. *n* = 5 per pollen load treatment. Treatment with >500 pollen grains did not result in any silique and are therefore not included. Data is described by non-linear functions, A) y = 28.99–587.9x^-1^ (inverse, solid line), *P* = 0.030 or y = 6.378 + 0.166x – 0.0025x^2^ (quadratic, dashed line), *P* = 0.033, and B) y = 95.37 – 1647x ^-1^ (inverse, solid line), *P* = 0.047. The straight lines refer to mean values in control silique of Compass (CH), where dotted = open pollinated from common garden, dash-dotted = autonomously selfed in greenhouse, and dash-dot-dotted pooled for greenhouse and common garden (as no differences were found between environments for this trait).

In the controlled hand-pollination experiment with cv. Compass (CH), the smallest pollen load (25 grains) resulted in seed set in 1 out of 5 pollinations (Fig 1). For the pollen load treatments between 50 and 500 pollen grains, silique formation occurred in 2–4 out of 5 pollinations (Fig 1). No seeds were produced at the highest pollen load in any of the 5 pollinations (> 500 pollen grains), and this was therefore not included in Fig 1. Despite a high variation in seed set, both the number of seeds per siliqua and seed weight per siliqua could be described by an increasing function with diminishing returns, i.e. the increase was highest at low pollen load sizes and then levelled off at about 100–200 pollen grains (Fig 1A and 1B). Number of seeds per siliqua could also be explained by a quadratic function (Fig 1A), suggesting a decrease in number of seeds for higher pollen load sizes (500 pollen grains). In line with the other data on number of seeds per siliqua and seed weight per siliqua (see Statistics section), the two seed traits were strongly correlated (Pearson *r* = 0.670, df = 17, *P* = 0.0017). Neither number of seeds nor seed weight per siliqua following hand-pollinations differed from that in control silique in Compass (from both greenhouse and common garden) in a pairwise comparison to each of the pollination load sizes (from 50 to 500 pollen grains, Dunnet t-tests; *P* > 0.084, Fig 1A and 1B).

### Developmental rate and plant height at the time of potting in cultivars and feral populations

At the time of potting, the seven oilseed rape winter cultivars did not differ in developmental stage compared to the five feral populations. However, cultivars were generally higher than ferals (cultivars: 35.0 ± 1.62 cm (mean ± s.e.), ferals: 22.6 ± 2.48 cm), Nested ANOVA; Category of plant (cultivar vs. feral): *F*_1,11.1_ = 2.51, *P* = 0.14, Cultivar/Population (Category of plant): *F*_10,121_ = 8.87, *P* < 0.001).

### Pollen germination rate and pollen tube growth rate in cultivars and feral populations

**Fig 2:**
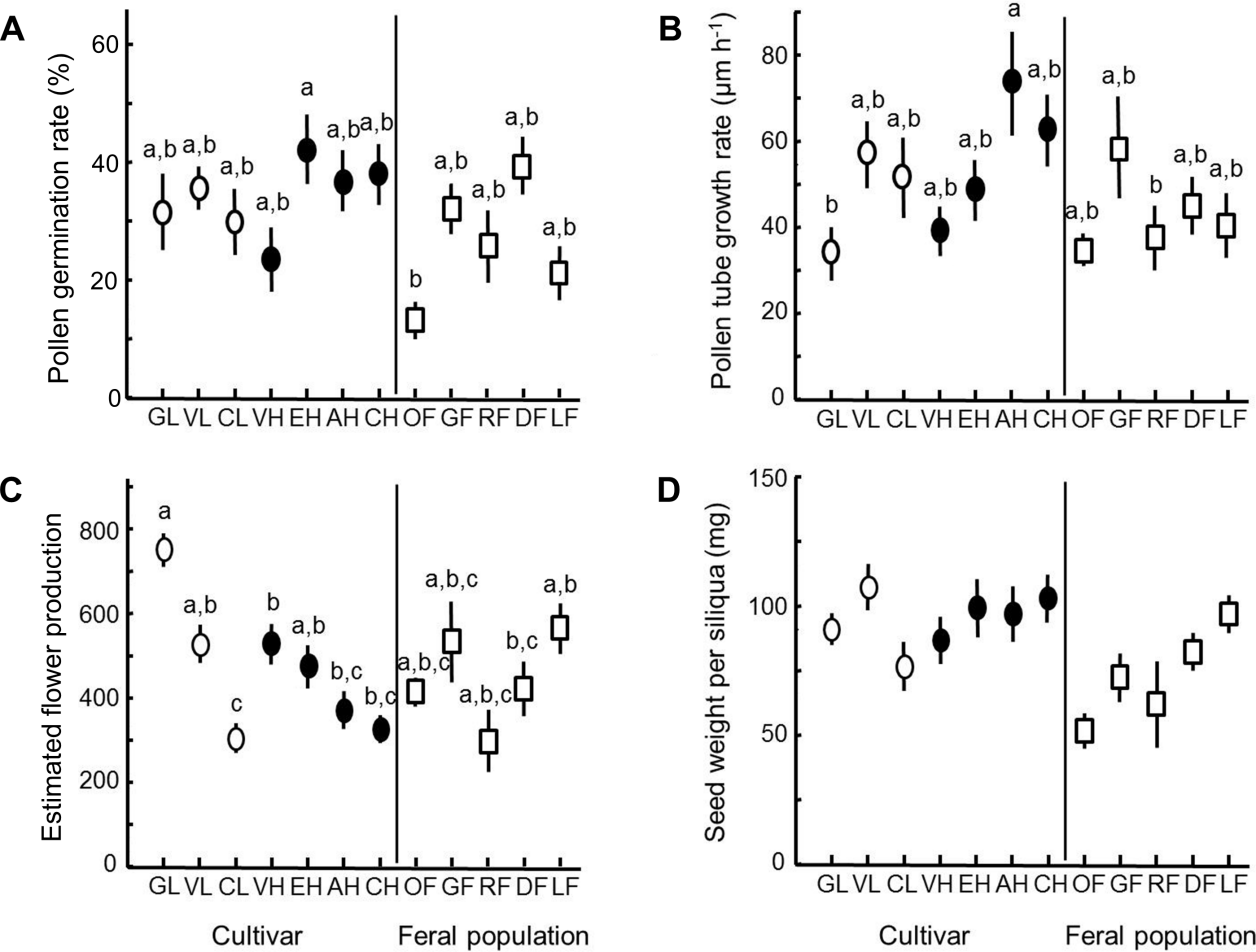
Variation in estimated traits in oilseed rape winter cultivars and feral populations. **(A)** Pollen germination rate *in vitro*, **(B)** pollen tube growth rate *in vitro*, **(C)** estimated flower production (= number of flowers on the main raceme times the number of branches), and **(D)** seed weight per siliqua. Most data were generated from plants grown in a common garden. For cultivars, data for C and D was pooled for plants grown in greenhouse and common garden, as no differences between growth conditions were found. Open circles = open-pollinated cultivars, filled circles = hybrid cultivars, open squares = feral populations. Error bars denote one standard error. Different letters above data points indicate significant differences in a posthoc test (Tukey HSD) involving both cultivated and feral plants (analysis 2). See S1 Table for an explanation of abbreviated cultivar/feral population codes.

In the seven oilseed rape winter cultivars included in our experiment, pollen tube growth rate *in vitro*, but not pollen germination rate, differed significantly (analysis 1, Table 1, Fig 2A and 2B). A posthoc-test (Tukey HSD) revealed that Abakus (AH) had significantly higher pollen tube growth rate than Visby (VH) and Galileo (GL) (Fig 2B). No general difference between open-pollinated or hybrid cultivars was detected. Pollen tube growth rate was strongly positively correlated to pollen germination rate (Table 1).

**Table 1:** Nested ANOVA (analysis 1) for in vitro pollen traits, estimated flower production and seed weight per siliqua in oilseed rape winter cultivars (either open pollinated or hybrid bred), all grown in a common garden.

Source of variation	Pollen germination rate			Pollen tube growth rate (μm h^-1^)			Estimated flower production			Seed weight per siliqua (mg)		
	df	*F*	*P*	df	*F*	*P*	df	*F*	*P*	df	*F*	*P*
Pollen germination rate				**1**	**17.4**	**0.0001**						
Cultivar (Breeding method)	5	1.36	0.26	**5**	**2.92**	**0.023**	**5**	**14.4**	**< 0.0001**	5	0.965	0.45
Breeding method	1	0.429	0.52	1	0.982	0.33	**1**	**10.4**	**0.002**	1	0.35	0.56
Error	46			45			95			59		

Different cultivars are nested under breeding method. Data for estimated flower production and seed weight is pooled for plants grown in greenhouse and common garden, as no differences between growth condition were found. Pollen germination rate is used as a covariate in the model for pollen tube growth rate. Significant values are presented in bold. Pollen germination rate = percentage germinated pollen, rescaled as an arcsine-transformed proportion. Estimated flower production = number of flowers in main raceme times the number of branches.

We could not detect any significant difference between the seven winter oilseed rape cultivars and five feral populations for either pollen germination rate or pollen tube growth rate (analysis 2, Table 2, Fig 2A and 2B). However, pollen germination rate was significantly influenced by plant category, i.e. which cultivar/feral population the plant belonged to (Table 2, Fig 2A and 2B). In a posthoc analysis (Tukey HSD), we detected significant differences between the cultivar Excalibur (EH) and the feral population Örja (OF) (see Fig 2A). Thus, only for this specific combination the grown cultivar could be shown to have significantly higher pollen germination rate than the feral population. For pollen tube growth rate we found a similar trend that the identity of cultivar/feral population was important, but the effect was marginally non-significant (*P* = 0.059; Table 2, Fig 2B). This trait was rather highly affected by the covariate pollen germination rate (Table 2), which was in line with analysis 1 involving only the seven cultivars. Posthoc tests showed no significant differences among feral populations for any of the pollen traits.

**Table 2:** Nested ANOVA (analysis 2) for in vitro pollen traits, estimated flower production and seed weight per siliqua in oilseed rape winter cultivars and feral populations (= category of plant), all grown in a common garden.

Source of variation	Pollen germination rate			Pollen tube growth rate (μm h^-1^)			Estimated flower production			Seed weight per siliqua (mg)		
	df	*F*	*P*	df	*F*	*P*	df	*F*	*P*	df	*F*	*P*
Pollen germination rate				**1,70**	**16.5**	**0.0001**						
Cultivar/Pop (Category)	**10,71**	**2.15**	**0.031**	10,70	1.90	0.059	**10,120**	**9.0**	**< 0.0001**	10,84	1.42	0.19
Category of plant	1,10.92	0.053	0.11	1,11.79	0.429	0.53	1,11.1	0.078	0.79	**1,13.2**	**7.54**	**0.016**

Different cultivars/feral populations (random effect) are nested under category of plant. For estimated flower production and seed weight per silique in cultivars, data is pooled for plants grown in greenhouse and common garden, as no difference between growth condition was found. For feral populations, all data is from common garden. Pollen germination rate is used as a covariate in the model for pollen tube growth rate. df denotes the numerator and denominator degrees of freedom, respectively. Significant values are presented in bold. Pollen germination rate = percentage germinated pollen, rescaled as an arcsine-transformed proportion. Estimated flower production = number of flowers in main raceme times the number of branches.

### Nectar production in cultivars

Nectar production in the seven greenhouse-grown oilseed rape cultivars was generally very low (mean volume ± s.e.: 0.031 ± 0.0035 μl, *n* = 50). We were unable to detect any significant differences among the cultivars (Kruskal-Wallis; H (48, *n* = 50) = 48.8, *P* = 0.44).

### Components of flower and seed production in cultivars and feral populations

Breeding method had an impact on estimated flower production, suggesting that open-pollinated cultivars generally produced more flowers compared to hybrids when calculated as number of flowers on the main raceme times number of branches (analysis 1, Table 1, see Fig 2C). However, within open-pollinated cultivars there were significant differences in estimated number of flowers. In particular, the cultivar Galileo (GL) showed a high estimated flower production (Fig 2C), indicating that the detected difference between breeding methods was driven by this cultivar. In the analysis taking both cultivated and feral plants into account (analysis 2), estimated flower production was strongly affected by cultivar/feral population nested within category of plant (cultivated or feral), but there was no difference between these two categories (Table 2, Fig 2C). Posthoc tests further suggested only significant differences among cultivars but not among feral populations.

Seed weight per siliqua was unaffected by both cultivar and breeding method (analysis 1, Table 1). However, seed weight per siliqua was significantly higher in cultivated plants compared to feral plants, as suggested by the significant effect of category of plant (analysis 2, Table 2, Fig 2D). Likewise, the mean seed mass of an individual seed was higher in cultivars (5.25 ± 0.50 g) than in ferals (3.72 ± 0,54 g) (Mixed model ANOVA; *F*_1, 96_ = 23,88, *P* < 0,001). Seed weight per siliqua did not differ among cultivars/feral populations (Table 2, Fig 2D).

## Discussion

### Seed quantity and seed weight per siliqua in relation to pollen load size in the hybrid cultivar Compass

Despite recent interest in insect pollination in agricultural crops [2,41,42], we often lack fundamental knowledge of pollination biology, e.g. how large pollen loads are needed for full seed set. Even though our hand-pollination experiment in the hybrid Compass showed a lot of variability, the relationship between pollen load size and seed quantity could be described by a typical increasing function with diminishing returns that levelled off at about 100–200 pollen grains (Fig 1A), suggesting the end of the filling stage [14]. The fit was almost as good for a quadratic relationship indicating that larger pollen load size could result in lowered seed set. Moreover, no seeds were produced for crosses with the largest pollen load size (> 500 pollen grains). Reduced seed set for larger pollen loads could be caused by pollen clogging of the stigma [15] or seed abortion, which is common in this species [43].

The relationship between pollen load size and seed weight per siliqua was very similar to that for number of seeds per siliqua (Fig 1B), but the relationship for seed weight did not indicate a significant reduction for larger pollen load sizes. Under our experimental conditions we could not find any evidence that our measure of seed quality, i.e. seed weight per siliqua, increased due to higher levels of pollen competition in the pistil (sorting stage, [14], which is known from other species (e.g., [44,45]). On the other hand, we used only two pollen donors in our crosses, limiting the probability to sample superior fathers when several pollen donors contribute to the pollen load [46].

Our result is relatively consistent with the only previous study we have found that investigated the relationship between pollen load size and seed quantity per siliqua in oilseed rape. By counting pollen grains on the stigmatic surface in a cage experiment with pollinators, a linear relationship was detected for up to 150 pollen grains before levelling off in the open pollinated cultivar Jet-neuf [47c]. Field estimates of the number of pollen grains accumulating on the stigma in oilseed rape was shown to vary with pollinator abundance, from 150 pollen grain per hour to 150 pollen grains after five days [32]. Another study showed that a single bumble-bee visit resulted in a mean of 141 pollen grains on the stigma in flowers with most self-pollen removed [48], but a mean of 547 pollen grains when self-pollen contributed to the deposition. Thus, it appears that pollen limitation is not a common occurrence in oilseed rape. In future studies it would be of interest to investigate if varieties differ in how much pollen is needed for full seed set and if this is modified by the presence of self pollen, which could influence the benefit of pollination. In particular, it would be important to evaluate differences between open pollinated and hybrid cultivars, to test whether such differences could explain detected differences in benefits of insect pollination [5].

### Variability in traits with potential effects on pollination success in winter oilseed rape cultivars

Recent studies suggest that winter oilseed rape cultivars differ in yield benefit from insect pollination, and that open-pollinated cultivars can be particularly responsive to higher pollination intensity compared to hybrids [4,5]. In the present study, we assessed variability in pollen viability in a competitive environment, nectar production, and components of flower- and seed production among both open-pollinated and hybrid cultivars, to identify traits that potentially cause variability in yield due to differences in insect-pollination dependence. In contrast to nectar and seed production per siliqua, pollen tube growth rate *in vitro*, and flower production estimated as number of flowers on the main raceme times the number of branches showed significant variability among cultivars (Fig 2, Table 1). Estimated flower production, but not pollen viability differed significantly between cultivar type; the three open-pollinated cultivars had higher mean estimated flower production than the four hybrids (Fig 2C). However, it should be noted that the difference between cultivar types mainly was driven by the cultivar Galileo that showed the highest estimated flower production, indicating that the difference was rather dependent on cultivar than breeding method. Similarly, Carruthers et al. [8] also showed high variation in total flower production and floral nectar among cultivars rather than differences between open pollinated and hybrid cultivars with restored male sterility. While a high number of flowers on the main raceme, which is one component of our estimate of flower production, may be positively related to an early seed set and longer seed filling period, we need more direct estimates of this trait and pollinator influence on seed yield to determine if flower production can explain some of the detected cultivar differences in benefits of insect pollination [4,5].

### Comparison between winter oilseed rape cultivars and feral populations

Gene flow in oilseed rape is known to occur over long distances [16,19], and in particular information on variability in pollen traits is useful to assess pollen dispersal between cultivated fields as well as among feral or wild populations. Because ferality has been shown to cause negative effects in the Brassicaceae *Raphanus raphanistrum*, leading to for example earlier flowering and absence of a swollen root [49], we hypothezised that pollen viability, seed size and seed mass per siliqua would be lower in ferals than in cultivars. Even though our results showed reduced seed weight per individual seed and reduced seed weight per siliqua in ferals (Fig 2D, Table 2), there were no overall difference in pollen traits (Fig 2A and 2B) or estimated flower production (Fig 2C) between cultivars and feral plants, suggesting that ferality did not decrease these traits. However, we cannot exclude that these traits would have been inferior in plants grown in field margins, that presumably are more nutrient poor than cultivated fields (cf. [37]), than in our potted experimental plants.

In the present study, only one cultivar had higher pollen germination rate than one of the feral populations (Fig 2B). To the extent that our measure of pollen viability influences fertilization success and gene flow, this result suggests that the probability of gene flow from ferals to cultivated plants will depend on which cultivars and which feral populations are in contact. Moreover, the low number of pollen grains needed for seed set, as implied from our crossing experiment in the cultivar Compass (Fig 1), suggests that some gene flow may occur under a broad range of conditions (e.g. from small feral populations and in years with unfavourable weather for pollinating insects) as seeds can be formed already when very few pollen grains are transferred to stigmas. A more comprehensive study with a range of *B*. *napus* cultivars is needed to elucidate differences between hybrid and open-pollinated cultivars as well as between individual cultivars and ferals.

Since the processes involved in gene flow from oilseed rape are known to be very variable, both plant traits and crop management are likely to affect gene flow [17]. Recently, cultivar was suggested to be more important than management for survival of volunteer *B*. *napus* in agricultural fields [50]. The generally lower seed weight per siliqua and per individual seed detected in our feral populations could affect the likelihood of establishment of these plants, potentially as a consequence of smaller seeds leading to smaller plants with reduced ability to cope with herbivory and competition as previously suggested for *B*. *napus* [26]. A difference in starting conditions in our study (ferals had a lower stature than crop plants at the start of the experiment) may have affected seed weight per siliqua, but more likely the shorter stature in ferals was due to an observed higher allocation to roots than an overall difference in biomass. However, germination tests need to be carried out to establish if the seeds with lower biomass also result in poorer germination and poorer seedling performance.

## Conclusions

In this study, both the seed quantity and seed weight per siliqua were saturated at relatively low pollen load sizes. Thus, seed production per siliqua in at least the tested cultivar may be less sensitive to low levels of pollen receipt, exemplifying how basic knowledge concerning the pollination process could add to our understanding of insect pollination in crops. We also detected differences in pollen traits and estimated flower production among seven cultivars. These traits could contribute to the detected differences in yield among oilseed rape cultivars, and should be taken into consideration when exploring differences in pollination requirements among cultivars. Provided that pollen viability is of importance for fertilization success and gene flow, our finding that pollen viability can be high despite an indication of reduced seed weight per silica in ferals–in combination with the low pollen load needed for seed set—implies that even small feral populations may contribute to seed set of cultivated plants. Pollen load studies on other cultivars as well as ferals are needed to establish if this is a general pattern, especially if open-pollinated and hybrid cultivars differ in pollen load size needed for seed set. Moreover, we need a better understanding of the implications of reduced seed weight per silica in ferals, e.g. in relation to establishment, competition and herbivory. In future studies increased understanding of the traits influencing pollination success and gene flow in insect-pollinated crops could contribute to improve plant breeding, decisions about suitable cultivars, crop management as well as increase awareness of gene flow in the agro-ecological interface.

## Supporting information

S1 Appendix Trait comparisons between common garden and greenhouse environments, and repeatability and age effects in pollen trait estimates. (DOCX)

S1 Supporting data file Plant traits. (XLSX)

S2 Supporting data file Pollen load experiment. (XLSX)

S3 Supporting data file Pollen traits. (XLSX)

S4 Supporting data file Pollen traits all measurements. (XLSX)

S5 Supporting data file Pollen age effects. (XLSX)

S6 Supporting data file Nectar content. (XLSX)

S7 Supporting data file Seed traits. (XLSX)

S1 Table Oilseed rape winter cultivars and feral populations (abbreviations within brackets) collected either from cultivated fields or field margins in early spring in south-west Scania, as specified by coordinates. (DOCX)

S2 Table ANOVA (mixed model) for proportion germinated pollen and pollen tube growth rate in vitro repeatedly measured 4–8 times under varying environmental conditions in six individual plants of oilseed rape (plant ID, random effect), all grown in a common garden. (DOCX)
