# Supplementary material for: Variable pollen viability and effects of pollen load size on components of seed set in cultivars and feral populations of oilseed rape
Source: PLoS One. 2018 Sep 20;13(9):e0204407. doi: 10.1371/journal.pone.0204407 (PMC6147549; doi:10.1371/journal.pone.0204407)
Supplement: S1 Appendix — (DOCX) [file pone.0204407.s001.docx]

S1 Appendix. Trait comparisons between common garden and greenhouse environments, and repeatability and age effects in pollen trait estimates

**Trait variation in cultivars in common garden and greenhouse**

In order to understand how much of the estimated variability of plant traits in cultivars was likely to change across environments, we compared plants grown in the greenhouse with plants from the common garden with respect to pollen traits, components of flower-, and seed production (Table 1). Apart from abiotic differences between environments, flowers produced by plants in the common garden were open pollinated, i.e. had the opportunity for both self and outcross pollination, while flowers produced by plants in the greenhouse only had the opportunity for autonomous self-pollination.

Plants of all seven cultivars grown in the much warmer greenhouse generally showed largely reduced pollen germination (mean ± s.d.: 0.19 ± 0.12), as opposed to in the common garden (0.35 ± 0.15), overriding any differences among cultivars (Two-way ANOVA; Growth condition: *F*_1,81_ = 28.6, *P* < 0.0001, Cultivar: *F*_6,81_ = 1.27, *P* = 0.28, Interaction: *F*_6,81_ = 0.521, *P* = 0.79). Pollen tube growth rate was not directly affected by growth condition, but was influenced by the effect of pollen germination (Two-way ANOVA; Proportion pollen germination (covariate): *F*_1,80_ = 14.5, *P* = 0.0003, Growth condition: *F*_1,80_ = 3.68, *P* = 0.058, Cultivar: *F*_6,80_ = 1.67, *P* = 0.14, Interaction: *F*_6,80_ = 1.76, *P* = 0.12).

Estimated flower production was significantly influenced by cultivar (Two-way ANOVA; *F*_6,94_ = 13.5, *P* < 0.001), but not by growing in the common garden or the greenhouse (*F*_1,94_ = 0.118, *P =* 0.73). Seed weight per siliqua in the six cultivars grown in both the open-pollinated common garden and the insect-free greenhouse (Table 1) was independent of growing conditions (Two-way ANOVA; *F*_1,58_ = 0.451, *P =* 0.51) and cultivar (*F*_6,58_ = 0.972, *P* = 0.45). Number of seeds per siliqua, on the other hand, was significantly affected by growing conditions (Two-way ANOVA; *F*_1,58_ = 11.7, *P* = 0.001) but not by cultivar (*F*_6,58_ = 1.039, *P =* 0.41), with higher number of seeds produced in the common garden (mean ± s.e.: 25.0 ± 2.33, *n* = 41) than in the greenhouse (17.9 ± 0.62, *n* = 18).

**Repeatability, environmental influence and age effects in pollen measurements**

To get an indication of repeatability of pollen trait measurements among individual plants (suggesting a genetic influence on this trait), some plants from the common garden were germinated repeatedly (in total six plants from three cultivars and three feral populations were germinated 4-8 times). To be able to evaluate the effect of environmental factors on these repeated measurements we noted temperature in the growth chamber (varied between 19.5 and 21.5 ºC) and outdoor weather condition (measured as greenhouse temperature ranging between 23 and 30 ºC, as this temperature was strongly related to weather categorized as i) rainy/overcast, ii) partly cloudy, iii) sunny; *P* < 0.0001).

Additionally, during separate pollen germinations we investigated the effect of pollen age on pollen traits by repeatedly germinating pollen from newly opened flower (ca one day old) and pollen from older flowers (all anthers open) in the same plant individuals. We used ten individual plants (five cultivated and five feral plants from the common garden).

For repeated measurements of pollen traits, we performed mixed model ANOVA, including plant individual (random factor), and temperature of growth chamber and outdoor weather condition (measured as temperature of greenhouse) (continuous covariates) and the two-way interactions between plant ID and continuous covariates, respectively. For the model with pollen tube growth rate as dependent variable, we also included pollen germination rate as a continuous covariate.

Pollen tube growth rate was significantly influenced by both plant individual and by the slight temperature difference (2 ºC) in the growth chamber in the repeated measurements (Table S2), but not by outdoor weather condition. This result indicates high repeatability for pollen tube growth rate among individual plants despite an environmental effect of the temperature during pollen tube growth. Pollen tube growth rate was also affected by the marginally significant (*P* = 0.048) interaction between plant individual and temperature in the germination chamber (Table S2), implying that plants are differently affected by this temperature. Pollen germination rate was not significantly affected by either plant individual or any of the environmental factors (Table S2). However, as in previous analyses pollen tube growth rate was strongly positively influenced by pollen germination rate (Table S2), potentially suggesting some repeatability for pollen germination rate among plants.

Age of pollen did not influence pollen tube growth rate (Paired *t*-test; df = 9, *P* = 0.75), but there was a tendency that younger pollen had a lower pollen germinationrate than older pollen (mean ± s.d.: 46 ± 24 % vs. 29 ± 9 %, Paired *t*-test; df = 9, *P* = 0.089).
