# Supplementary material for: Variable pollen viability and effects of pollen load size on components of seed set in cultivars and feral populations of oilseed rape
Source: PLoS One. 2018 Sep 20;13(9):e0204407. doi: 10.1371/journal.pone.0204407 (PMC6147549; doi:10.1371/journal.pone.0204407)
Supplement: S1 Table — (DOCX) [file pone.0204407.s009.docx]

**S1 Table. Oilseed rape winter cultivars and feral populations (abbreviations within brackets) collected either from cultivated fields or field margins in early spring in south-west Scania, as specified by coordinates**

| **Source** | **Cultivar** | **Breeding method** | **Feral population** |  | **Coordinates** | | **Experiment/estimated trait** | |
| --- | --- | --- | --- | --- | --- | --- | --- | --- |
|  |  |  |  | ***n*** | **X** | **Y** | **Greenhouse** | **Common garden** |
| Cultivation | Galileo (GL) | OP |  | 16 | 6176104 | 1339534 | B, C, D, E | B, D |
| Cultivation | Vision (VL) | OP |  | 14 | 6175839 | 1338186 | B, C, D, E | B, D, E |
| Cultivation | Catalina (CL) | OP |  | 15 | 6179361 | 1330146 | B, C, D, E | B, D, E |
| Cultivation | Visby (VH) | Hybrid |  | 15 | 6198352 | 1317770 | B, C, D, E | B, D, E |
| Cultivation | Excalibur (EH) | Hybrid |  | 14 | 6198352 | 1317770 | B, C, D, E | B, D, E |
| Cultivation | Abakus (AH) | Hybrid |  | 14 | 6198352 | 1317770 | B, C, D, E | B, D, E |
| Cultivation | Compass (CH) | Hybrid |  | 15 | 6198352 | 1317770 | A, B, C, D, E | B, D, E |
| Field margin |  |  | Örja (OF) | 4 | 6198394 | 1316964 |  | B, D, E |
| Field margin |  |  | Gastelyckan (GF) | 5 | 6176344 | 1338734 |  | B, D, E |
| Field margin |  |  | Dalbyrondell (RF) | 6 | 6172917 | 1344288 |  | B, D, E |
| Field margin |  |  | Dalbyvägen (DF) | 9 | 6175344 | 1341084 |  | B, D, E |
| Field margin |  |  | Lundadäck (LF) | 7 | 6175719 | 1340459 |  | B, D, E |

Breeding method of cultivars was either open pollinated (OP) or hybrid. *n* = number of plants. Plants were grown either in a greenhouse or a common garden. A = Pollen load size experiment, B = Pollen germination rate and pollen tube growth rate *in vitro*, C = Nectar production (secretion volume), D = Estimated flower production, E = Seed production per siliqua.
