# Supplementary material for: Variable pollen viability and effects of pollen load size on components of seed set in cultivars and feral populations of oilseed rape
Source: PLoS One. 2018 Sep 20;13(9):e0204407. doi: 10.1371/journal.pone.0204407 (PMC6147549; doi:10.1371/journal.pone.0204407)
Supplement: S2 Table — (DOCX) [file pone.0204407.s010.docx]

**S2 Table. ANOVA (mixed model) for proportion germinated pollen and pollen tube growth rate in vitro repeatedly measured 4-8 times under varying environmental conditions in six individual plants of oilseed rape (plant ID, random effect), all grown in a common garden**

| **Source of variation** | **Pollen germination rate** | | | **Pollen tube growth rate (µm h^-1^)** | | |
| --- | --- | --- | --- | --- | --- | --- |
|  | **df** | ***F*** | ***P*** | **df** | ***F*** | ***P*** |
| Pollen germination rate |  |  |  | **1** | **5.70** | **0.027** |
| Temperature growth chamber | 1 | 1.94 | 0.17 | **1** | **5.66** | **0.028** |
| Outdoor weather condition | 1 | 2.41 | 0.13 | 1 | 0.149 | 0.70 |
| Plant ID | 5 | 1.66 | 0.18 | **5** | **3.32** | **0.025** |
| Temp GC × Plant ID | - | - | - | **5** | **2.78** | **0.048** |
| Outdoor weather × Plant ID | - | - | - | 5 | 1.94 | 0.14 |
| Error | 30 |  |  | 19 |  |  |

As an indication of environmental influence on response traits we noted temperature variation in the growth chamber and outdoor weather condition (as indicated by estimates of greenhouse temperature). The two latter traits are used as covariates in the models. Pollen germination rate is included as a covariate in the model for pollen tube growth rate. Non-significant interactions (*P* > 0.20) were removed from the model. Significant values are presented in bold. Pollen germination rate = percentage germinated pollen, rescaled as an arcsine-transformed proportion.
